# Cross-Sectional Study on Attitudes among General Practitioners towards Pneumococcal Vaccination for Middle-Aged and Elderly Population in Hong Kong

**DOI:** 10.1371/journal.pone.0078210

**Published:** 2013-11-05

**Authors:** Lancelot W. H. Mui, Alvin Y. S. Chan, Albert Lee, John Lee

**Affiliations:** 1 JC School of Public Health and Primary Care, Faculty of Medicine, The Chinese University of Hong Kong, Hong Kong SAR, China; 2 The Hong Kong Medical Association, Hong Kong SAR, China; Rockefeller University, United States of America

## Abstract

**Objective:**

To study the attitudes among general practitioners towards pneumococcal vaccination for middle-aged (50–64) and elderly population (over 65) in Hong Kong and the factors affecting their decision to advise pneumococcal vaccination for those age groups.

**Design:**

Cross-sectional study of general practitioners in private practice in Hong Kong.

**Participants:**

Members of Hong Kong Medical Association delivering general practice services in private sector.

**Measuring Tool:**

Self-administered questionnaire.

**Main Outcome Measures:**

Intention to recommend pneumococcal vaccination, barriers against pneumococcal vaccination.

**Results:**

53.4% of the respondents would actively recommend pneumococcal vaccination to elderly patients but only 18.8% would recommend for middle-aged patients. Consultation not related to pneumococcal vaccine was the main reason for not recommending pneumococcal vaccine (43.6%). Rarity of pneumonia in their daily practice was another reason with 68.4% of respondents attending five or less patients with pneumonia each year. In multivariate analysis, factors such as respondents would get vaccination when reaching age 50 (OR_m_ 10.1), and attending 6 pneumonia cases or more per year (OR_m_ 2.28) were found to be associated with increasing likelihood for recommending vaccination to the middle-aged. While concerns of marketing a product (OR_m_ 0.41), consultation not related to vaccination (OR_m_ 0.45) and limited time (OR_m_ 0.38) were factors that reduced the likelihood.

**Conclusion:**

Public policy is needed to increase the awareness of impact of pneumococcal pneumonia and the availability of preventive measures.

## Introduction

Pneumonia is the third leading cause of mortality in Hong Kong, with over 6,000 mortalities in the Year 2010 [1]. World-wide, pneumococus is responsible for more deaths than any other single pathogen [2]. It disproportionately affects the older population, especially those who are over 65 years old [3]. Pneumococcal pneumonia is preventable through vaccination [4]. Studies have also found early evidence for pneumococcal vaccination in reducing the incidence of myocardial infarction [5] and ischemic stroke [6]. In Australia, there is a growing concern with the rapid aging population (also applicable to other developed part of the world including Hong Kong), and the mortality rates in elderly due to Streptococcus pneumoniae would exceed 20% even with the best available antibiotic treatment [7]. Moreover, one ought to be more cautious with increasing antibiotic resistance among many strains of pneumococcus.

Currently there are two types of vaccines available in Hong Kong for pneumococcal vaccination: a 23-valent pneumococcal polysaccharides vaccine (PPV23) [8] and a 13-valent pneumococcal conjugate vaccine (PCV13) [9], both of which are indicated for use in adults age 50 or above by the US FDA. A 10-valent pneumococcal conjugated vaccine (PCV10) is also available in some other countries. Currently, Hong Kong Government only recommends pneumococcal vaccination for those who are 65 years of age or above [10]. As a result, free or subsidized pneumococcal vaccination for this high risk group is available through the “Government Vaccination Programme” and “Elderly Vaccination Subsidy Scheme” (EVSS).

For middle-aged people of 50–64 years, vaccination through private healthcare providers is their only option. In order to prepare general practice for pandemic influenza, planned, practised and habitual infection controls such as annual influenza immunisation and pneumococcal immunisation for at-risk people are recommended [11]. Lack of recommendation by their general practitioners (GPs) and holding a belief that vaccine can cause illness or symptoms were main reasons for refusal of pneumococcal vaccine reported in a qualitative study [12]. In addition, knowledge about the availability and purpose of the pneumococcal vaccine was also found to be poor. This suggests that beside appropriate education campaigns, it is important for GPs to develop a trusting and positive relationships with their patients to improve the likelihood of immunisation uptake.

The goal of this study is to study the attitudes among general practitioners towards pneumococcal vaccination for middle-aged and elderly population in Hong Kong and the factors affecting their decision to advise pneumococcal vaccination for those age groups as they play a significant role in communicating the risks and benefits to patients.

## Materials and Methods

### Ethics statement

The purpose of the study was explained to the physicians in the mailing. Consent to participate was implied by returning the questionnaire to the research team. This study has been approved by the Survey and Behavioural Research Ethics Committee of The Chinese University of Hong Kong.

### Study design and sampling method

This is a cross-sectional study of GPs who are in private practice in Hong Kong as majority of GPs are in private practice in Hong Kong [13]. Physicians from the Hong Kong Medical Association were contacted through its membership database and a structured self-administered questionnaire was mailed to 3,792 private physicians in February 2013. They were asked whether they are providing GP services and only those practitioners delivering GP services were included for analysis. Response to the questionnaire was tracked through a unique serial number which was not used for any other purpose. A second mailing to non-respondents was conducted in April 2013 to increase the response rate. Other measures to increase response rate included stamped return envelope for the questionnaire.

### Questionnaire

The questionnaire included questions on basic demographics, intention to advise pneumococcal vaccination, patient composition, years of clinical experience, field of practice, clinical setting, participation in the government's vaccination subsidy scheme, vaccination status of the physicians themselves, and barriers against advising pneumococcal vaccination. The questionnaire was developed by an expert panel of public health researchers and physicians to ensure good face and content validity.

### Statistical analysis

Statistical analysis was conducted using PASW Statistics version 18. Descriptive statistics were calculated for all questions in the questionnaire. Factors associated with pneumococcal vaccine recommendation were identified through binary logistic regression. Univariate logistic regressions were conducted for association between pneumococcal vaccine recommendations with each of the independent variables. Independent variables tested to be significant were then tested in a multivariate model and a parsimonious model was obtained by stepwise backward elimination.

## Results and Discussion

### A representative sample of GPs in Hong Kong

Response rate to the study after two rounds of mailings was 24.7% (937 out of 3792). Seventy six of the returned questionnaires were not include for the analysis because they were either incomplete or the respondents had indicated that they were not currently active in clinical practice (retirement, no clinic, or not actively practicing). Therefore, data from 861 respondents were included for data analysis. The age of this sample (mean  = 55.91, SD  = 12.63) is not significantly different from the age of the complete list in the database (mean  = 56.76, SD  = 14.40; Student's t-test: p>0.05). Therefore, the study captured a representative sample of GPs in Hong Kong. Their characteristics are shown in Table 1.

**Table 1:** Respondents' characteristics.

	n	% or Mean (SD)
Age (years)	724	55.91 (12.63)
Gender		
Male	680	79.5%
Female	175	20.5%
Missing	6	
Major field of practice		
General Practice / Family Medicine	531	61.7%
Internal Medicine	62	7.2%
Paediatrics	56	6.5%
Surgery	39	4.5%
Others	172	20.0%
Missing	1	
Medical experience (years)	858	32.6 (12.72)
Setting of primary clinical practice		
Solo practice	666	77.5%
Group / Corporate practice	144	16.8%
Private hospital	26	3.0%
NGO	13	1.5%
Others	10	1.2%
Missing	2	
Geographical location of the clinic		
Hong Kong Island	296	34.5%
Kowloon	347	40.4%
New Territories	215	25.1%
Missing	3	
Received influenza vaccination last year	519	61.6%
Missing	18	
Patient composition (age 50–64)		
0–25%	216	26.4%
25–50%	387	47.3%
50–75%	199	24.3%
75–100%	17	2.1%
Missing	42	
Patient composition (age 65+)		
0–25%	410	53.5%
25–50%	242	31.6%
50–75%	95	12.4%
75–100%	20	2.6%
Missing	94	
Number of pneumonia patient in past year		
None	256	31.2%
1–5	305	37.2%
6–10	164	20.0%
11+	95	11.6%
Missing	41	

### Current practice on pneumococcal vaccination recommendation

Table 2 shows that nearly half (45.0%) of the respondents indicated that they intended to (or had already) receive pneumococcal vaccine when reaching age 50 or above. Also nearly half (45.3%) worked in a clinic participating in the EVSS. These two variables show a significant statistical association (Pearson's chi-square: p<0.001; data not shown). However it is not possible to state the causative effect between the two factors and the temporality of the relationship is also unknown because of the cross-sectional nature of this study.

**Table 2:** Practice and attitudes towards pneumococcal vaccination.

	n	% or Mean (SD)
Intend to receive PV* at age 50 or above		
Yes	385	45.0%
No	217	25.4%
Uncertain	254	29.7%
Missing	5	
Clinic participating in EVSS*		
Yes	385	45.3%
No	464	54.7%
Missing	12	
Actively advise PV* to patients (65 years or above)		
Yes	453	53.4%
No	267	31.4%
Uncertain	129	15.2%
Missing	12	
Actively advise PV* to patients (50–64 years)		
Yes	150	18.8%
No	433	54.1%
Uncertain	217	27.1%
Missing	61	
Barriers to recommend PV* to indicated patients		
Not related to consultation	375	43.6%
Patients have low perceived severity	351	40.9%
Perceived to be marketing	288	33.4%
Affordability of the vaccine	247	28.8%
Patients worry about safety	224	26.1%
Patients do not concern about preventive healthcare	213	24.7%
Rare disease among own patients	211	24.6%
Limit consultation time	156	18.2%
Reluctance among patients due to incomplete protection	82	9.5%
Others	68	7.9%
Total number of barriers	861	2.57 (1.62)
Government's role†		
Government should increase subsidy for EVSS*	691	83.5%
Missing	33	
Government should NOT expand EVSS* coverage to all patients aged 50+	316	39.5%
Missing	60	
Government should promote severity of pneumonia as part of its pneumococcal vaccination promotion	763	91.8%
Missing	30	

* PV: Pneumococcal Vaccine; EVSS: Elderly Vaccination Subsidy Scheme

† Percentage who answered “Strongly agree” or “Agree”

While 53.4% of the respondents stated that they would actively recommend pneumococcal vaccination to patients aged 65 or above, only 18.8% would recommend for those who are 50–64 years old (Table 2). This difference in recommendation practice might be because currently the Hong Kong Government only recommends pneumococcal vaccine to people aged 65 or above. If fact, only a minority of the respondents (39.5%) indicated that the government should not expand EVSS coverage to patients aged 50 or above. Therefore, extension of recommendation to those aged 50 or above would be an effective way to promote pneumococcal vaccination. Publicly-funded vaccination program would have impact on vaccination. A study in Australia showed that pneumococcal vaccination rates increased from 7% to 51% within 2 years of such programme [14].

Consultation not related to pneumococcal vaccine was the most frequently-cited reason as the main barrier for not recommending pneumococcal vaccine to eligible patients (43.6%). Although many countries recommend that opportunistic preventive care, such as vaccination, is recommended during consultation [15], [16], [17], our results did not show such practice in Hong Kong. One possible reason would be the concern about perception of marketing medical product, with 33.4% of respondents expressing such thought. It is important that patients can rely upon the independence and trustworthiness of advice or treatment offered by medical professionals [18]. If doctors are not sensitive to the potential influence of pharmaceutical industry on their prescribing behaviour, this would have ethical failing on independence and trustworthy doctor-patient relationship [19]. Similar findings were shown in a study for Human Papillomavirus (HPV) Vaccine for cervical cancer prevention, which showed that GPs worried about hard selling of an expensive vaccine [20]. Another reason might be the rarity of pneumonia patients presenting to GPs. Among the respondents, 68.4% reported that they attended five or less patients with pneumonia each year, and 24.6% indicated that this would be a deterrent for them to recommend pneumococcal vaccine. The general practice morbidity survey in 2007–08 also showed that less than 1% of GP consultation was due to pneumonia [21].

Low perception of severity about pneumonia by patients was another major barrier for GPs to recommend pneumococcal vaccine (40.9%; Table 2), even though pneumonia is the third leading cause of death in Hong Kong [1]. A study reviewing evidence of behaviour change in patients identified three main factors: self monitoring of health behaviours, risk communication and making use of social supports [22]. Risk communication from GPs and public health authority would have impact on shaping positive health behaviours such as uptake of vaccination. Social support and self-monitoring of health behaviours leading to positive behaviours such as vaccination would be difficult with general lack of preventive healthcare concern among patients, as indicated by 24.7% of the GP respondents. The vast majority of the respondents (91.8%; Table 2) indicated that the government should emphasise the severity in its pneumococcal vaccine promotion strategy to the public to raise their awareness. Lack of integration at policy level and inadequate infrastructure were found to be the main barriers of health promotion by GPs, apart from patients' compliance and attitudes as well as attitudes of GPs towards health promotion [23].

Nearly one out of three (28.8%) respondents indicated that pneumococcal vaccine is too expensive for their patients, and 83.5% of the respondents thought that the government should increase the amount of subsidy for the EVSS (Table 2). Studies of HPV vaccination also found cost to be a major barrier [20], [24]. The combined effect of increase in subsidy, along with the expansion to those who are aged 50 or above, should help to boost the vaccination rate, as study by Keating et al. found that inadequate reimbursement from insurance can be a main obstacle for uptake of expensive vaccine [24].

Patients' concern of vaccine safety would deter GPs to advise for vaccination as reflected by 26.1% of the respondents. However, majority of the respondents did not think that time limitation during consultation and reluctance among patient because of incomplete protection would affect their decision to recommend pneumococcal vaccine (Table 2). Physician should be the key influence for vaccination uptakes as a study revealed that recommendation by the physician was the main reason for being vaccinated and lack of information as the most important reason for not being vaccinated [25]. GPs could make use of modern techniques of health promotion in motivating patients to adopt preventive health practice [23].

### Factors associated with recommendation of pneumococcal vaccine to the middle-aged and the elderly

Results from multivariate logistic regressions (Table 3) show that the factors associated with recommending pneumococcal vaccine to middle-aged (50–64 years old) and elderly (65+ years old) share some commonalities but differ in some key aspects. The strongest factor associated with pneumococcal vaccine recommendation, especially for the middle-aged, is whether respondent would get pneumococcal vaccination when reaching age 50: recommend to middle-aged (OR_m_ = 10.104, p<0.001); recommend to elderly (OR_m_ = 3.628, p<0.001). This result is expected as they teach what they preach. High uptake rate of vaccination by GPs could be a more effective way to promote pneumococcal vaccination to general population in Hong Kong. If medical practitioners offer and advocate vaccination to their patients, then a significant majority of patients would accept it, even though the patient has a negative attitude towards vaccination [26], [27]. Physicians would cause under-use if they are themselves reluctant and not convinced about safety, efficacy and need of influenza and pneumococcal vaccinations [26]. The leading cause of missed opportunities for vaccination is the failure of physicians to offer it [27]. This approach has strong theoretical support from the Diffusion of Innovations model [28], under which adoption of an innovation (i.e. pneumococcal vaccination) in a society can be sped up by utilising opinion leaders and change agents (i.e. the GPs) in the society.

**Table 3:** Multivariate logistic regression for factors associated with recommending pneumococcal vaccination to middle-aged and elderly patients.

	50–64 years old patients		65+ years old patients	
	OR_m_^§^	95% CI	OR_m_^§^	95% CI
Major field of practice				
General Practice / Family Medicine	—		1	
Internal Medicine	—		0.850	(0.446, 1.620)
Paediatrics	—		0.751	(0.367, 1.538)
Surgery	—		0.624	(0.261, 1.490)
Others	—		0.442	(0.260, 0.752)^ †^
Setting of primary clinical practice				
Solo practice	¶		—	
Group / Corporate practice	¶		—	
Private hospital	¶		—	
NGO	¶		—	
Others	¶		—	
Received influenza vaccination last year				
No	—		1	
Yes	—		1.441	(0.994, 2.090)
Number of pneumonia patient in past year				
None	1		1	
1–5	1.696	(0.953, 3.018)	1.980	(1.250, 3.137)^ †^
6+	2.277	(1.281, 4.048)^ †^	1.702	(1.035, 2.798)*
Receive pneumococcal vaccine at 50				
No / Uncertain	1		1	
Yes	10.104	(6.057, 16.854)^ ‡^	3.628	(2.486, 5.295)^ ‡^
Participating in EVSS				
No	—		1	
Yes	—		2.296	(1.557, 3.387)^ ‡^
Number of barriers	—		¶	
Barriers to advise of PV				
Affordability	¶		—	
Rare disease among patients	—		0.447	(0.289, 0.690)^ ‡^
Lack of concern about prevention	—		¶	
Low perceived severity	—		1.382	(0.945, 2.021)
Safety concern by patients	—		1.542	(1.019, 2.333)*
Concern about incomplete protection	¶		¶	
Marketing	0.406	(0.249, 0.664)^ ‡^	0.391	(0.267, 0.571)^ ‡^
Limited consultation time	0.337	(0.178, 0.639)^ †^	¶	
Unrelated to consultation	0.454	(0.285, 0.721)^ †^	0.644	(0.453, 0.916)*

* p<0.05; ^†^p<0.01; ^‡^p<0.001; ^§^Multivariate odds ratio; ^¶^Variable not included in multivariate model because it was not significant in the univariate model; — variable removed from multivariate model by backward stepwise elimination; Other variables tested include age, gender, location of clinic, patient composition and were not statistically significant in univariate analysis for both patient age groups (p>0.05).

On the other hand, although there is a significant association between whether respondent would get pneumococcal vaccination when reaching age 50 and whether they received influenza vaccine last year (Pearson's chi-square p-value<0.001; data not shown), receiving influenza vaccination was not associated with recommendation of pneumococcal vaccine to either age group (p>0.05, Table 3). This suggests that GPs' decision to recommend pneumococcal vaccine was not guided by their general attitude towards vaccination (as indicated by whether they received influenza vaccine).

Number of pneumonia patient managed affected the respondents' decision to recommend pneumococcal vaccine to their patients, especially when the number is high (more than 6 per year) (Table 3). The perceived susceptibility construct from the Health Belief Model appears to be at work here [29]. Instead of influencing behaviour that affects the respondents themselves, it affected their behaviour (recommending vaccine) towards others.

Concern about promoting vaccine not related to the consultation and the concerns of marketing a medical product were found to be the barriers that negatively affected recommendation to both middle-aged and elderly patients (Table 3). The effects were similar for concern about marketing (OR_m_ = 0.406 and 0.391, p<0.001 and p<0.001, respectively for middle-aged and elderly). However, the effect from concern about promoting vaccine in unrelated consultation was more potent for the middle-aged group (OR_m_ = 0.454, p<0.01) than for the elderly (OR_m_ = 0.644, p<0.05). The delivery of preventive services in GPs' offices usually falls below recommended level [30], and the ethical concerns of marketing a product would make it even worse. The basic principles underlying the conduct of doctors with respect to pharmaceutical products and companies should be openness and transparency [31]. Availability of evidence-based findings from academic and professional bodies and recommendation policy from government would resolve the dilemma. GPs might not view health promotion programs as worthwhile and are not very familiar with latest model of health promotion linking to holistic approach of patient care [23], [32]. To address the gap, two areas of GP training need significant amendments: the undergraduate curriculum for MBBS students and continuing medical education (CME) for graduates and established GPs [23], [33].

Interestingly, respondents who indicated that patients' concern about pneumococcal vaccine safety deterring them from making recommendation were positively associated with actual recommendation to the elderly (OR_m_ = 1.542, p<0.05; Table 3), but not to the middle-aged (p>0.05). One possible explanation would be that the doctors felt obligated to talk to patients who had safety concern, and ended up advising the patient to take the pneumococcal vaccine as it is in line with public health policy. Study on healthy eating and promotion of physical activities has shown the importance of developing public policies to foster supportive environment conducive to healthy behaviours [34]. However, since pneumococcal vaccination for the middle-aged is still not recommended by public policy, the doctors might hesitate in recommending to the middle-aged as the environment is not seen to be supportive for vaccination of this target group.

Participation in the EVSS is significantly association with recommendation to elderly (OR_m_ = 2.296, p<0.001; Table 3) but not the middle-aged (p>0.05). This is expected as the EVSS only covers the elderly group. However, the doctors are not only driven by monetary incentive as evidence from the study findings that if pneumococcal disease is rare among their elderly patients, the doctors were less likely to recommend vaccination (OR_m_ = 0.447, p<0.001). A Swiss study has reported GPs mentioning low priority of the pneumococcal vaccination in daily practice as they rarely experienced case of severe pneumococcal disease in their daily work [35]. The GPs also perceived insufficient evidence resulting from existing epidemiologic data and clinical trials that enhanced the little attention given to the pneumococcal vaccination. It becomes more important to expand the coverage scheme for the middle-aged so GPs would appreciate their significant role in delivering preventive health services.

### Government is pivotal to remove barriers to recommend pneumococcal vaccine and GPs would provide trustworthy communication on prevention

From the results, the government need to lay down policy on pneumococcal vaccination for middle-aged. Acceptance and adoption of preventive interventions by the individuals who comprise society is dependent on the honesty, knowledge, understanding and skill of information providers [36]. A study investigating the differences in attitudes, knowledge and belief of hospital health workers and community doctors for vaccination of elderly has shown that physicians' barriers were patient's refusal and competing priorities [37]. Those barriers are particularly important for hospital health care doctors who were less likely to regard vaccination as a priority [37]. Although hospital health care workers encounter far more pneumonia cases, they might not view recommendation for vaccination to be accorded high priority. GPs were found to be more likely to support vaccination among the elderly in the study [37]. Our findings also show that GPs would fulfill the role and they would be provided further training in delivery of effective health promotion practice [23]. The government's policy and recommendations would help shape the behaviours of the GPs in private practice, who handles around 70% of primary care in Hong Kong [13]. The government could also increase the coverage of the EVSS to include all pneumococcal vaccines with proven efficacy. Efforts are also needed to increase the epidemiologic data on the pneumococcal vaccination available to both GPs and public. Lancet paper by Marmot et al. argued for health care being a common good and not a market commodity when health care systems have the best outcomes when based in primary health care [38]. It becomes a mandate for countries to invest in primary health care, especially for preventive health care such as vaccination, as GPs could facilitate uptake rate even when vaccination would be free of charge [39].
